# Rates of *ICD-10* Code U09.9 Documentation and Clinical Characteristics of VA Patients With Post–COVID-19 Condition

**DOI:** 10.1001/jamanetworkopen.2023.46783

**Published:** 2023-12-08

**Authors:** Pandora L. Wander, Aaron Baraff, Alexandra Fox, Kelly Cho, Monika Maripuri, Jacqueline P. Honerlaw, Yuk-Lam Ho, Andrew T. Dey, Ann M. O’Hare, Amy S. B. Bohnert, Edward J. Boyko, Matthew L. Maciejewski, Elizabeth Viglianti, Theodore J. Iwashyna, Denise M. Hynes, Thomas F. Osborne, George N. Ioannou

**Affiliations:** 1 Division of General Internal Medicine, Veterans Affairs (VA) Puget Sound Health Care System and University of Washington, Seattle; 2 Seattle Epidemiologic Research and Information Center, VA Puget Sound Health Care System, Seattle, Washington; 3 VA Centralized Interactive Phenomics Resources–VA Boston Healthcare System, Boston, Massachusetts; 4 Department of Medicine, Mass General Brigham, Harvard Medical School, Boston, Massachusetts; 5 Division of Nephrology, VA Puget Sound Healthcare System and University of Washington, Seattle; 6 VA Center for Clinical Management Research, Ann Arbor, Michigan; 7 Department of Psychiatry, University of Michigan Medical School, Ann Arbor; 8 Center of Innovation to Accelerate Discovery and Practice Transformation, Durham VA Health Care System, Durham, North Carolina; 9 Department of Population Health Sciences, Duke University School of Medicine, Durham, North Carolina; 10 Duke-Margolis Center for Health Policy, Duke University School of Medicine, Durham, North Carolina; 11 Division of General Internal Medicine, Duke University School of Medicine, Durham, North Carolina; 12 Center for Clinical Management Research, VA Ann Arbor Health System, Ann Arbor, Michigan; 13 Division of Pulmonary and Critical Care Medicine, Department of Internal Medicine, University of Michigan, Ann Arbor; 14 Center of Innovation to Improve Veteran Involvement in Care, VA Portland Health Care System, Portland, Oregon; 15 Health Management and Policy, School of Social and Behavioral Health Sciences, College of Public Health and Human Sciences, Health Data and Informatics Program, Center for Genome Research and Biocomputing, Oregon State University, Corvallis; 16 VA Palo Alto Health Care System, Palo Alto, California; 17 Department of Radiology, Stanford University School of Medicine, Stanford, California; 18 Division of Gastroenterology, VA Puget Sound Health Care System and University of Washington, Seattle; 19 Research and Development, VA Puget Sound Health Care System, Seattle, Washington

## Abstract

**Importance:**

A significant proportion of SARS-CoV-2 infected individuals experience post–COVID-19 condition months after initial infection.

**Objective:**

To determine the rates, clinical setting, risk factors, and symptoms associated with the documentation of *International Statistical Classification of Diseases Tenth Revision* (*ICD-10*), code U09.9 for post–COVID-19 condition after acute infection.

**Design, Setting, and Participants:**

This retrospective cohort study was performed within the US Department of Veterans Affairs (VA) health care system. Veterans with a positive SARS-CoV-2 test result between October 1, 2021, the date *ICD-10* code U09.9 was introduced, and January 31, 2023 (n = 388 980), and a randomly selected subsample of patients with the U09.9 code (n = 350) whose symptom prevalence was assessed by systematic medical record review, were included in the analysis.

**Exposure:**

Positive SARS-CoV-2 test result.

**Main Outcomes and Measures:**

Rates, clinical setting, risk factors, and symptoms associated with *ICD-10* code U09.9 in the medical record.

**Results:**

Among the 388 980 persons with a positive SARS-CoV-2 test, the mean (SD) age was 61.4 (16.1) years; 87.3% were men. In terms of race and ethnicity, 0.8% were American Indian or Alaska Native, 1.4% were Asian, 20.7% were Black, 9.3% were Hispanic or Latino, 1.0% were Native Hawaiian or Other Pacific Islander; and 67.8% were White. Cumulative incidence of U09.9 documentation was 4.79% (95% CI, 4.73%-4.87%) at 6 months and 5.28% (95% CI, 5.21%-5.36%) at 12 months after infection. Factors independently associated with U09.9 documentation included older age, female sex, Hispanic or Latino ethnicity, comorbidity burden, and severe acute infection manifesting by symptoms, hospitalization, or ventilation. Primary vaccination (adjusted hazard ratio [AHR], 0.80 [95% CI, 0.78-0.83]) and booster vaccination (AHR, 0.66 [95% CI, 0.64-0.69]) were associated with a lower likelihood of U09.9 documentation. Marked differences by geographic region and facility in U09.9 code documentation may reflect local screening and care practices. Among the 350 patients undergoing systematic medical record review, the most common symptoms documented in the medical records among patients with the U09.9 code were shortness of breath (130 [37.1%]), fatigue or exhaustion (78 [22.3%]), cough (63 [18.0%]), reduced cognitive function or brain fog (22 [6.3%]), and change in smell and/or taste (20 [5.7%]).

**Conclusions and Relevance:**

In this cohort study of 388 980 veterans, documentation of *ICD-10* code U09.9 had marked regional and facility-level variability. Strong risk factors for U09.9 documentation were identified, while vaccination appeared to be protective. Accurate and consistent documentation of U09.9 is needed to maximize its utility in tracking patients for clinical care and research. Future studies should examine the long-term trajectory of individuals with U09.9 documentation.

## Key Points

**Question:**

What are the rates, risk factors, clinical settings, and symptoms associated with documentation of the *International Statistical Classification of Diseases, Tenth Revision*, code U09.9 for post–COVID-19 condition?

**Findings:**

In this cohort study of 388 980 US veterans with positive test results for SARS-CoV-2 during the Omicron era (October 1, 2021, to January 31, 2023), 5% had U09.9 documentation by 12 months after infection. Patterns varied by geographic location and clinical setting; risk factors included older age, female sex, Hispanic or Latino ethnicity, hospitalization within 30 days of the first positive SARS-CoV-2 test result, receipt of mechanical ventilation, lack of vaccination, and a higher number of symptoms at presentation.

**Meaning:**

Accurate and consistent documentation of U09.9 is needed to maximize its utility in tracking patients for clinical care and research.

## Teaser

This cohort study assesses the rates, risk factors, clinical setting, and symptoms associated with the documentation of *International Statistical Classification of Diseases, Tenth Revision* (*ICD-10*) code U09.9 for post–COVID-19 condition among US veterans with positive test results for SARS-CoV-2.

## Introduction

More than 3 years after the World Health Organization declared COVID-19 a pandemic, post–COVID-19 condition (PCC) has emerged as one of its most enduring manifestations, affecting as many as 65 million people worldwide.^1^ Post–COVID-19 condition^2^ may present with a multitude of debilitating symptoms, such as fatigue, shortness of breath, and cognitive dysfunction. More than 200 symptoms and conditions have been described that are present 4 weeks or more after the initial infection, involving multiple organ systems,^3^ including pulmonary, cardiovascular, cerebrovascular, thromboembolic, neurocognitive, mental health, metabolic, kidney, and gastrointestinal tract systems.^4,5,6,7,8,9,10,11,12,13^ The etiology of PCC is unclear, but possible causes include viral persistence, autoimmunity triggered by infection and molecular mimicry, reactivation of latent viruses (such as Epstein-Barr virus and human herpesvirus 6), effects of SARS-CoV-2 on microbiota, dysfunctional signaling in the brainstem and/or vagus nerve, microvascular blood clotting with endothelial dysfunction, and inflammation-triggered chronic changes leading to organ damage.^1^ Multiple different phenotypes of PCC likely exist, which may correlate with different causes listed above, with a broad severity spectrum.

Limited information is available about which patients seek care for manifestations of PCC, the extent to which clinicians document care as management of PCC, or who is providing such care. These factors contribute to challenges in estimating the incidence and prevalence of PCC. An *International Statistical Classification of Diseases, Tenth Revision* (*ICD-10*) code for PCC became available in the US on October 1, 2021, which affords the possibility of investigating the correlates of documentation of PCC care by clinicians. We examined the rates, risk factors, clinical settings, and symptoms associated with the documentation of *ICD-10* code U09.9 among persons with positive test results for SARS-CoV-2 in the national US Veterans Affairs (VA) health care system from October 1, 2021, until January 31, 2023.

## Methods

### Data Source and Study Population

The VA is the largest integrated national health care system in the US, providing care at 172 medical centers throughout the country. We used data from the VA’s Corporate Data Warehouse^14^ and the COVID-19 Shared Data Resource (CSDR), which include analytic variables on all VA enrollees who received testing for SARS-CoV-2, derived from the VA’s comprehensive electronic health records (EHR) system.^15^

We identified all VA enrollees who had documentation in the VA EHR of their first positive SARS-CoV-2 RNA polymerase chain reaction or antigen test result in a respiratory specimen between October 1, 2021, and January 31, 2023 (n = 411 837), which corresponded to an Omicron variant period after the end of the Delta period in October and November 2021. We excluded 22 857 individuals who did not have at least 1 primary care encounter in the VA in the 18 months before receiving a positive test result, providing an analytic cohort of 388 980 individuals. The study was approved by the VA Puget Sound Institutional Review Board, which waived the requirement to obtain informed consent. The study followed the Strengthening the Reporting of Observational Studies in Epidemiology (STROBE) reporting guideline.

### Outcome Ascertainment

The study’s outcome was defined as documentation of the *ICD-10* code U09.9 for post–COVID-19 condition, unspecified, after the test-positive date. We identified this code in records for all outpatient and inpatient encounters in the VA health care system using the Corporate Data Warehouse. To get the most complete ascertainment of U09.9 documentation possible, we also identified whether this code was documented in 2 non-VA health care sources as follows. First, we used detailed claims data from the VA Community Care program, which coordinates and reimburses VA purchased care provided in the community. Second, we obtained claims data from the Centers for Medicare & Medicaid Services (CMS) for care received by veterans enrolled in VA health care outside the VA health care system, provisioned by the VA Information Resource Center, the availability of which extended only to September 2022 at the time of analysis.

### Baseline Characteristics

We ascertained sociodemographic, geographic, and clinical characteristics (including comorbid conditions and medication prescriptions based on a 2-year lookback window prior to infection) that were potentially associated with U09.9 documentation (Table 1). The *ICD-10* codes used to define each comorbid condition were provided by the VA Centralized Interactive Phenomics Resource.^16^ We determined whether primary vaccination was administered (ie, 2 doses of messenger RNA-1273 [Moderna], 2 doses of BNT162b2 [Pfizer-BioNTech] or a single dose of Ad26.COV2.S [Janssen]) or at least 1 booster dose was administered prior to the date of infection. In addition to vaccinations administered by VA, CSDR captures some COVID-19 vaccines given outside the VA (eg, pharmacies, health departments, mass vaccination centers, and clinics) and electronically reported to the VA or documented by VA clinicians. To further improve COVID-19 vaccination ascertainment, we additionally captured vaccinations administered through CMS-Medicare and the VA Community Care program.

**Table 1.** Baseline Sociodemographic Characteristics and Association With Documentation of *ICD-10* Code U09.9

Characteristic	No. (%) of patients			Cumulative incidence of U09.9 documentation per 100 persons (95% CI)		HR (95% CI)	
	All	Documentation of code U09.9					
		No	Yes	6 mo	12 mo	Crude	Adjusted^a^
All patients	388 980 (100)	370 393 (100)	18 587 (100)	4.79 (4.72-4.86)	5.28 (5.20-5.35)		
Age, y^b^							
18-49	97 505 (25.1)	93 563 (25.3)	3942 (21.2)	3.86 (3.74-3.99)	4.28 (4.15-4.41)	1 [Reference]	1 [Reference]
50-59	67 524 (17.4)	64 389 (17.4)	3135 (16.9)	4.50 (4.34-4.66)	5.03 (4.86-5.20)	1.18 (1.12-1.23)	1.26 (1.20-1.32)
60-64	41 956 (10.8)	40 048 (10.8)	1908 (10.3)	4.50 (4.30-4.71)	4.97 (4.74-5.19)	1.18 (1.12-1.25)	1.27 (1.20-1.34)
65-69	40 540 (10.4)	38 574 (10.4)	1966 (10.6)	4.88 (4.66-5.10)	5.42 (5.18-5.66)	1.28 (1.21-1.35)	1.30 (1.23-1.38)
70-74	59 714 (15.4)	56 574 (15.3)	3140 (16.9)	5.40 (5.21-5.59)	5.88 (5.67-6.08)	1.40 (1.34-1.47)	1.43 (1.36-1.51)
75-79	44 660 (11.5)	42 317 (11.4)	2343 (12.6)	5.51 (5.28-5.73)	6.04 (5.80-6.29)	1.45 (1.38-1.53)	1.47 (1.39-1.56)
80-84	17 160 (4.4)	16 217 (4.4)	943 (5.1)	5.83 (5.45-6.20)	6.52 (6.10-6.94)	1.56 (1.45-1.67)	1.50 (1.39-1.62)
85-89	11 496 (3.0)	10 804 (2.9)	692 (3.7)	6.57 (6.08-7.06)	7.16 (6.62-7.70)	1.75 (1.62-1.90)	1.60 (1.47-1.74)
≥90	8424 (2.2)	7906 (2.1)	518 (2.8)	7.07 (6.47-7.67)	7.39 (6.76-8.02)	1.89 (1.73-2.08)	1.67 (1.52-1.84)
Sex							
Men	339 522 (87.3)	323 344 (87.3)	16 178 (87.0)	4.80 (4.72-4.87)	5.27 (5.19-5.35)	1 [Reference]	1 [Reference]
Women	49 458 (12.7)	47 049 (12.7)	2409 (13.0)	4.76 (4.57-4.96)	5.29 (5.08-5.50)	0.99 (0.95-1.04)	1.23 (1.18-1.29)
Race							
American Indian or Alaska Native	3267 (0.8)	3076 (0.8)	191 (1.0)	5.95 (5.09-6.79)	6.52 (5.61-7.43)	1.14 (0.99-1.32)	1.08 (0.93-1.24)
Asian	5477 (1.4)	5284 (1.4)	193 (1.0)	3.59 (3.09-4.10)	3.75 (3.22-4.27)	0.67 (0.58-0.78)	0.93 (0.80-1.07)
Black or African American	80 673 (20.7)	77 918 (21.0)	2755 (14.8)	3.43 (3.30-3.56)	3.74 (3.60-3.88)	0.66 (0.63-0.69)	0.72 (0.69-0.75)
Native Hawaiian or Pacific Islander	3936 (1.0)	3738 (1.0)	198 (1.1)	4.90 (4.20-5.59)	5.49 (4.73-6.24)	0.97 (0.84-1.11)	0.94 (0.81-1.08)
White	263 684 (67.8)	250 143 (67.5)	13 541 (72.9)	5.15 (5.07-5.24)	5.68 (5.59-5.78)	1 [Reference]	1 [Reference]
Declined or missing	31 943 (8.2)	30 234 (8.2)	1709 (9.2)	5.34 (5.08-5.59)	5.92 (5.64-6.20)	1.03 (0.98-1.09)	0.97 (0.92-1.02)
Ethnicity							
Hispanic or Latino	36 178 (9.3)	33 482 (9.0)	2696 (14.5)	7.39 (7.10-7.67)	8.48 (8.16-8.79)	1.65 (1.59-1.72)	1.33 (1.27-1.39)
Not Hispanic or Latino	329 505 (84.7)	314 665 (85.0)	14 840 (79.8)	4.52 (4.45-4.60)	4.94 (4.86-5.02)	1 [Reference]	1 [Reference]
Declined or missing	23 297 (6.0)	22 246 (6.0)	1051 (5.7)	4.51 (4.24-4.79)	4.97 (4.67-5.27)	1.00 (0.94-1.06)	0.96 (0.90-1.03)
Residence							
Rural	50 718 (13.0)	48 121 (13.0)	2597 (14.0)	5.13 (4.93-5.33)	5.64 (5.42-5.85)	1 [Reference]	1 [Reference]
Urban	338 262 (87.0)	322 272 (87.0)	15 990 (86.0)	4.74 (4.67-4.82)	5.22 (5.14-5.30)	0.91 (0.87-0.95)	0.90 (0.86-0.94)
VISN region							
8^c^	39 341 (10.1)	38 111 (10.3)	1230 (6.6)	3.11 (2.93-3.29)	3.39 (3.20-3.58)	1 [Reference]	1 [Reference]
7	22 875 (5.9)	22 237 (6.0)	638 (3.4)	2.78 (2.56-3.00)	3.05 (2.81-3.28)	0.89 (0.81-0.98)	1.07 (0.97-1.18)
16^c^	22 517 (5.8)	21 880 (5.9)	637 (3.4)	2.80 (2.57-3.02)	3.14 (2.89-3.38)	0.91 (0.83-1.00)	0.98 (0.89-1.08)
6	27 349 (7.0)	26 522 (7.2)	827 (4.4)	3.02 (2.81-3.23)	3.33 (3.10-3.55)	0.98 (0.89-1.07)	1.14 (1.04-1.24)
1	18 133 (4.7)	17 552 (4.7)	581 (3.1)	3.13 (2.87-3.39)	3.52 (3.24-3.81)	1.03 (0.94-1.14)	1.15 (1.04-1.28)
9	17 063 (4.4)	16 513 (4.5)	550 (3.0)	3.23 (2.96-3.51)	3.54 (3.25-3.84)	1.04 (0.94-1.15)	1.00 (0.90-1.11)
21^c^	24 544 (6.3)	23 713 (6.4)	831 (4.5)	3.32 (3.09-3.55)	3.72 (3.47-3.98)	1.09 (1.00-1.19)	1.17 (1.07-1.28)
2^c^	17 741 (4.6)	17 120 (4.6)	621 (3.3)	3.50 (3.21-3.78)	3.84 (3.54-4.14)	1.14 (1.03-1.25)	1.17 (1.06-1.29)
4	17 688 (4.5)	17 009 (4.6)	679 (3.7)	3.82 (3.53-4.11)	4.16 (3.85-4.47)	1.24 (1.13-1.36)	1.33 (1.21-1.46)
05^c^	13 491 (3.5)	12 951 (3.5)	540 (2.9)	3.97 (3.63-4.31)	4.40 (4.03-4.77)	1.30 (1.17-1.44)	1.37 (1.23-1.51)
15	14 177 (3.6)	13 605 (3.7)	572 (3.1)	4.14 (3.80-4.47)	4.32 (3.97-4.67)	1.30 (1.18-1.44)	1.26 (1.14-1.39)
22^c^	35 333 (9.1)	34 024 (9.2)	1309 (7.0)	3.72 (3.52-3.92)	4.10 (3.87-4.32)	1.20 (1.11-1.30)	1.22 (1.12-1.32)
23^c^	19 206 (4.9)	18 400 (5.0)	806 (4.3)	4.22 (3.93-4.52)	4.55 (4.23-4.86)	1.34 (1.23-1.47)	1.36 (1.24-1.49)
10	27 933 (7.2%)	26 738 (7.2)	1195 (6.4)	4.26 (4.02-4.51)	4.60 (4.34-4.86)	1.36 (1.26-1.47)	1.42 (1.31-1.54)
19^c^	19 014 (4.9)	18 062 (4.9)	952 (5.1)	5.05 (4.73-5.37)	5.37 (5.04-5.71)	1.61 (1.48-1.75)	1.65 (1.51-1.80)
12^c^	16 547 (4.3)	15 572 (4.2)	975 (5.2)	6.09 (5.71-6.47)	6.48 (6.08-6.88)	1.92 (1.77-2.09)	1.96 (1.80-2.13)
20^c^	14 187 (3.6)	13 284 (3.6)	903 (4.9)	6.39 (5.98-6.80)	6.71 (6.29-7.14)	2.07 (1.90-2.25)	2.22 (2.03-2.42)
17^c^	21 841 (5.6)	17 100 (4.6)	4741 (25.5)	21.92 (21.33-22.50)	24.90 (24.26-25.53)	7.41 (6.96-7.89)	7.60 (7.14-8.10)

Abbreviations: HR, hazard ratio; *ICD-10*, *International Statistical Classification of Diseases, Tenth Revision*; VISN, Veterans Integrated Service Network.

^a^ Adjusted by Cox proportional hazards regression for age (using the categories shown), sex, race, ethnicity, urban or rural residence, Charlson Comorbidity Index (CCI), and VISN. When we evaluated the associations of any of the individual comorbidities (eg, chronic obstructive pulmonary disease, congestive heart failure, chronic kidney disease, diabetes, depression, posttraumatic stress disorder, bipolar or schizoaffective disorder, cancer, hypertension, obesity, cerebrovascular disease, smoking, and others), we did not simultaneously adjust for the CCI because it would result in overadjustment.

^b^ Data were missing for 1 patient without U09.9 documentation.

^c^ Indicates facilities that have established dedicated clinics for the follow-up of patients with post–COVID-19 condition.

### Characteristics Related to the Severity of the Acute SARS-CoV-2 Infection

We ascertained 15 prespecified symptoms (eTable 1 in Supplement 1) present at the time of the positive test result or within the preceding 30 days, extracted from the EHR by a VINCI-CSDR natural language processing team using a combination of all relevant outpatient and inpatient clinical notes, which include COVID-19 symptom screening questionnaires, vital signs and relevant *ICD-10* codes for symptoms, when present. These symptoms could be related to COVID-19 but could also potentially be related to preexisting conditions. We identified whether SARS-CoV-2–infected persons were hospitalized in the VA health care system, CMS-Medicare, or the VA Community Care program within 30 days after a positive test result and among them those who underwent mechanical ventilation.

### Systematic Medical Record Review for Identification of New-Onset Symptoms in a Subset of Patients With U09.9 Documentation

Among all individuals in the VA health care system with a first instance of either a positive polymerase chain reaction test result or a U07.1 diagnosis code (March 1, 2020, to December 31, 2021), 350 individuals with a U09.9 code and at least 6 months of follow-up EHR documentation were chosen at random for systematic medical record review by 2 clinical adjudicators. The methods have been described in detail previously.^17^ Selected demographic characteristics of the primary study cohort and this subset are shown in eTable 2 in Supplement 1. Clinical notes were reviewed up to 6 months after acute COVID-19 infection, and only new-onset symptoms present for 30 days or more after acute COVID-19 infection were captured in accordance with the Centers for Disease Control and Prevention definition of post–COVID-19 conditions.^2^ Adjudicators had access to patient notes up to 1 year prior to their infection to assess any prior comorbidities and exacerbation of symptoms. This review effort has been previously described,^18,19^ but the results presented herein have not been published elsewhere.

### Statistical Analysis

In the primary study cohort, we evaluated whether characteristics at baseline and those related to the severity of the acute infection were associated with documentation of U09.9 using multivariable Cox proportional hazards regression with adjustment for age, sex, self-reported race and ethnicity (included as markers of racial and ethnic disparities), urban or rural residence (based on zip codes, using data from the VA Office of Rural Health,^20^ which uses the Secondary Rural-Urban Commuting Area for defining rurality), Charlson Comorbidity Index (CCI), and Veterans Integrated Service Network (VISN), the VA’s administrative regions.^21^ When we evaluated individual comorbidities, we did not simultaneously adjust for CCI to avoid overadjustment. Results are presented as crude hazard ratios (HRs) and adjusted HRs (AHRs) with a 95% CI. Time-to-event analyses began on the date of infection, and patients were censored on the date of death or at the end of follow-up on January 31, 2023.

## Results

### Characteristics of the Study Population

In our cohort of 388 980 patients with positive test results for SARS-CoV-2, 87.3% of patients were male and 12.7% were female; the mean (SD) age was 61.4 (16.1) years (46.8% aged ≥65 years).In terms of race, 0.8% of patients were American Indian or Alaska Native, 1.4% were Asian, 20.7% were Black, 1.0% were Native Hawaiian or Other Pacific Islander, and 67.8% were White); in terms of ethnicity, 9.3% of patients were Hispanic or Latino and 84.7% were non-Hispanic or non-Latino. The entire cohort had a high prevalence of comorbid conditions. During follow-up that extended to January 31, 2023, 18 587 patients (4.8%) had U09.9 documentation. Compared with patients without U09.9 documentation, those with U09.9 documentation were older, had a higher prevalence of multiple comorbid conditions (chronic obstructive pulmonary disease [COPD], congestive heart failure [CHF], chronic kidney disease [CKD], diabetes [primarily type 2]), higher CCI, higher hospitalization and ventilation rates, and more symptoms at the time of the acute SARS-CoV-2 infection (Table 1 and Table 2).

**Table 2.** Baseline Comorbidities, Medication Use, and SARS-CoV-2–Related Characteristics and Association With Documentation of *ICD-10* Code U09.9

Characteristic	No. (%) of patients			Cumulative incidence of U09.9 documentation per 100 persons (95% CI)		HR (95% CI)	
	All (n = 388 980)	Documentation of code U09.9					
		No (n = 370 393)	Yes (n = 18 587)	6 mo	12 mo	Crude	Adjusted^a^
**Comorbid conditions**							
CCI							
0	154 012 (39.6)	147 752 (39.9)	6260 (33.7)	3.95 (3.85-4.05)	4.37 (4.27-4.48)	1 [Reference]	1 [Reference]
1	79 049 (20.3)	75 166 (20.3)	3883 (20.9)	4.86 (4.70-5.02)	5.39 (5.22-5.56)	1.24 (1.20-1.30)	1.13 (1.09-1.18)
2	57 438 (14.8)	54 581 (14.7)	2857 (15.4)	5.06 (4.87-5.25)	5.54 (5.34-5.74)	1.28 (1.23-1.34)	1.12 (1.06-1.17)
3	33 561 (8.6)	31 741 (8.6)	1820 (9.8)	5.62 (5.36-5.87)	6.17 (5.89-6.45)	1.43 (1.36-1.51)	1.17 (1.10-1.23)
4	22 768 (5.9)	21 481 (5.8)	1287 (6.9)	5.89 (5.57-6.22)	6.53 (6.18-6.89)	1.52 (1.43-1.61)	1.15 (1.08-1.23)
5-6	24 829 (6.4)	23 402 (6.3)	1427 (7.7)	6.08 (5.76-6.39)	6.60 (6.26-6.95)	1.56 (1.47-1.65)	1.11 (1.05-1.18)
7-8	11 487 (3.0)	10 783 (2.9)	704 (3.8)	6.64 (6.15-7.13)	7.27 (6.73-7.80)	1.71 (1.58-1.85)	1.11 (1.02-1.20)
≥9	5836 (1.5)	5487 (1.5)	349 (1.9)	6.87 (6.15-7.58)	7.35 (6.57-8.11)	1.73 (1.56-1.93)	1.07 (0.96-1.20)
BMI^b^							
<18.5	4983 (1.3)	4711 (1.3)	272 (1.5)	6.07 (5.35-6.79)	6.47 (5.70-7.24)	1.31 (1.16-1.48)	0.95 (0.84-1.07)
18.5-25.0	70 911 (18.2)	67 636 (18.3)	3275 (17.6)	4.80 (4.63-4.96)	5.20 (5.02-5.38)	1 [Reference]	1 [Reference]
>25.0-30.0	130 882 (33.6)	124 950 (33.7)	5932 (31.9)	4.49 (4.37-4.61)	5.00 (4.88-5.13)	0.95 (0.91-0.99)	1.08 (1.03-1.12)
>30.0-35.0	104 388 (26.8)	99 393 (26.8)	4995 (26.9)	4.78 (4.65-4.92)	5.23 (5.08-5.37)	0.99 (0.95-1.04)	1.15 (1.10-1.20)
>35.0-40.0	49 123 (12.6)	46 557 (12.6)	2566 (13.8)	5.15 (4.94-5.35)	5.71 (5.49-5.93)	1.08 (1.03-1.14)	1.26 (1.19-1.32)
>40.0	26 867 (6.9)	25 363 (6.8)	1504 (8.1)	5.53 (5.25-5.82)	6.11 (5.80-6.41)	1.16 (1.09-1.23)	1.32 (1.24-1.41)
Diabetes							
No	265 482 (68.3)	253 568 (68.5)	11 914 (64.1)	4.44 (4.36-4.52)	4.91 (4.82-4.99)	1 [Reference]	1 [Reference]
Yes	123 498 (31.7)	116 825 (31.5)	6673 (35.9)	5.58 (5.44-5.71)	6.10 (5.96-6.25)	1.25 (1.22-1.29)	1.06 (1.02-1.09)
COPD							
No	328 833 (84.5)	313 979 (84.8)	14 854 (79.9)	4.49 (4.42-4.57)	4.96 (4.88-5.04)	1 [Reference]	1 [Reference]
Yes	60 147 (15.5)	56 414 (15.2)	3733 (20.1)	6.49 (6.28-6.70)	7.13 (6.90-7.35)	1.46 (1.41-1.52)	1.20 (1.16-1.25)
Asthma							
No	358 507 (92.2)	341 695 (92.3)	16 812 (90.5)	4.71 (4.63-4.78)	5.18 (5.10-5.26)	1 [Reference]	1 [Reference]
Yes	30 473 (7.8)	28 698 (7.7)	1775 (9.5)	5.81 (5.53-6.08)	6.41 (6.12-6.71)	1.24 (1.18-1.30)	1.26 (1.20-1.32)
CHF							
No	358 191 (92.1)	341 447 (92.2)	16 744 (90.1)	4.66 (4.58-4.73)	5.14 (5.06-5.22)	1 [Reference]	1 [Reference]
Yes	30 789 (7.9)	28 946 (7.8)	1843 (9.9)	6.48 (6.18-6.77)	7.00 (6.68-7.32)	1.40 (1.33-1.47)	0.96 (0.91-1.01)
Myocardial infarction							
No	378 596 (97.3)	360 611 (97.4)	17 985 (96.8)	4.76 (4.69-4.83)	5.24 (5.16-5.32)	1 [Reference]	1 [Reference]
Yes	10 384 (2.7)	9782 (2.6)	602 (3.2)	6.17 (5.67-6.66)	6.77 (6.23-7.31)	1.31 (1.21-1.42)	0.97 (0.89-1.05)
Cerebrovascular disease							
No	380 645 (97.9)	362 522 (97.9)	18 123 (97.5)	4.77 (4.70-4.84)	5.25 (5.18-5.33)	1 [Reference]	1 [Reference]
Yes	8335 (2.1)	7871 (2.1)	464 (2.5)	5.84 (5.30-6.37)	6.55 (5.95-7.14)	1.25 (1.14-1.37)	0.93 (0.85-1.02)
CKD							
No	335 559 (86.3)	320 128 (86.4)	15 431 (83.0)	4.57 (4.50-4.64)	5.04 (4.96-5.12)	1 [Reference]	1 [Reference]
Yes	53 421 (13.7)	50 265 (13.6)	3156 (17.0)	6.27 (6.05-6.49)	6.89 (6.65-7.12)	1.38 (1.33-1.44)	1.06 (1.02-1.11)
Peripheral arterial disease							
No	347 964 (89.5)	331 849 (89.6)	16 115 (86.7)	4.61 (4.54-4.69)	5.09 (5.01-5.16)	1 [Reference]	1 [Reference]
Yes	41 016 (10.5)	38 544 (10.4)	2472 (13.3)	6.39 (6.14-6.64)	7.02 (6.74-7.29)	1.40 (1.34-1.46)	1.07 (1.02-1.12)
Venous thromboembolism							
No	378 267 (97.2)	360 270 (97.3)	17 997 (96.8)	4.77 (4.70-4.84)	5.25 (5.17-5.32)	1 [Reference]	1 [Reference]
Yes	10 713 (2.8)	10 123 (2.7)	590 (3.2)	5.78 (5.31-6.25)	6.36 (5.84-6.87)	1.22 (1.13-1.33)	0.96 (0.89-1.05)
Obstructive sleep apnea							
No	257 494 (66.2)	245 820 (66.4)	11 674 (62.8)	4.54 (4.46-4.63)	5.01 (4.92-5.10)	1 [Reference]	1 [Reference]
Yes	131 486 (33.8)	124 573 (33.6)	6913 (37.2)	5.28 (5.16-5.41)	5.80 (5.66-5.94)	1.16 (1.13-1.20)	1.18 (1.14-1.21)
Obesity hypoventilation syndrome							
No	387 262 (99.6)	368 804 (99.6)	18 458 (99.3)	4.78 (4.71-4.85)	5.26 (5.19-5.34)	1 [Reference]	1 [Reference]
Yes	1718 (0.4)	1589 (0.4)	129 (0.7)	7.91 (6.54-9.26)	8.28 (6.85-9.68)	1.68 (1.41-1.99)	1.21 (1.02-1.44)
Depression							
No	249 187 (64.1)	237 630 (64.2)	11 557 (62.2)	4.67 (4.58-4.75)	5.13 (5.04-5.22)	1 [Reference]	1 [Reference]
Yes	139 793 (35.9)	132 763 (35.8)	7030 (37.8)	5.01 (4.89-5.13)	5.54 (5.41-5.66)	1.07 (1.04-1.10)	1.09 (1.06-1.13)
PTSD							
No	284 068 (73.0)	270 518 (73.0)	13 550 (72.9)	4.82 (4.73-4.90)	5.28 (5.19-5.37)	1 [Reference]	1 [Reference]
Yes	104 912 (27.0)	99 875 (27.0)	5037 (27.1)	4.73 (4.60-4.86)	5.27 (5.12-5.41)	0.99 (0.96-1.02)	1.04 (1.01-1.08)
Bipolar or schizoaffective disorder							
No	365 879 (94.1)	348 253 (94.0)	17 626 (94.8)	4.83 (4.76-4.91)	5.32 (5.24-5.40)	1 [Reference]	1 [Reference]
Yes	23 101 (5.9)	22 140 (6.0)	961 (5.2)	4.16 (3.89-4.43)	4.56 (4.27-4.85)	0.87 (0.81-0.92)	0.87 (0.82-0.93)
**Medications**							
Opioids							
No	366 338 (94.2)	349 010 (94.2)	17 328 (93.2)	4.73 (4.66-4.80)	5.22 (5.14-5.30)	1 [Reference]	1 [Reference]
Yes	22 642 (5.8)	21 383 (5.8)	1259 (6.8)	5.82 (5.49-6.13)	6.20 (5.86-6.54)	1.22 (1.15-1.29)	1.12 (1.05-1.19)
Antidepressants							
No	270 239 (69.5)	257 661 (69.6)	12 578 (67.7)	4.67 (4.59-4.76)	5.13 (5.04-5.22)	1 [Reference]	1 [Reference]
Yes	118 741 (30.5)	112 732 (30.4)	6009 (32.3)	5.06 (4.93-5.19)	5.61 (5.47-5.75)	1.09 (1.06-1.12)	1.12 (1.08-1.15)
Statins							
No	192 996 (49.6)	184 405 (49.8)	8591 (46.2)	4.38 (4.28-4.47)	4.83 (4.73-4.93)	1 [Reference]	1 [Reference]
Yes	195 984 (50.4)	185 988 (50.2)	9996 (53.8)	5.21 (5.11-5.32)	5.73 (5.62-5.84)	1.20 (1.16-1.23)	1.04 (1.01-1.08)
ACE inhibitors							
No	287 770 (74.0)	274 456 (74.1)	13 314 (71.6)	4.61 (4.53-4.69)	5.09 (5.00-5.17)	1 [Reference]	1 [Reference]
Yes	101 210 (26.0)	95 937 (25.9)	5273 (28.4)	5.33 (5.18-5.47)	5.83 (5.68-5.99)	1.15 (1.12-1.19)	1.01 (0.98-1.05)
ARBs							
No	330 485 (85.0)	314 936 (85.0)	15 549 (83.7)	4.70 (4.63-4.78)	5.17 (5.09-5.25)	1 [Reference]	1 [Reference]
Yes	58 495 (15.0)	55 457 (15.0)	3038 (16.3)	5.31 (5.12-5.51)	5.89 (5.68-6.09)	1.14 (1.10-1.19)	1.03 (0.99-1.07)
Calcium channel blockers							
No	291 130 (74.8)	277 461 (74.9)	13 669 (73.5)	4.67 (4.59-4.75)	5.16 (5.07-5.24)	1 [Reference]	1 [Reference]
Yes	97 850 (25.2)	92 932 (25.1)	4918 (26.5)	5.15 (5.00-5.29)	5.65 (5.49-5.81)	1.11 (1.07-1.15)	1.01 (0.98-1.05)
**COVID-19 vaccination**							
None	104 236 (26.8)	97 992 (26.5)	6244 (33.6)	5.92 (5.77-6.06)	6.44 (6.28-6.59)	1 [Reference]	1 [Reference]
Primary	134 603 (34.6)	128 007 (34.6)	6596 (35.5)	4.75 (4.63-4.86)	5.25 (5.12-5.37)	0.81 (0.78-0.83)	0.80 (0.78-0.83)
Booster	150 141 (38.6)	144 394 (39.0)	5747 (30.9)	4.03 (3.93-4.14)	4.44 (4.33-4.56)	0.68 (0.66-0.70)	0.66 (0.64-0.69)
**Indices of severity of acute SARS-CoV-2 infection**							
Hospitalization within 30 d of infection							
No	348 978 (89.7)	334 899 (90.4)	14 079 (75.7)	3.98 (3.92-4.05)	4.43 (4.36-4.51)	1 [Reference]	1 [Reference]
Yes	40 002 (10.3)	35 494 (9.6)	4508 (24.3)	12.46 (12.11-12.81)	13.42 (13.04-13.80)	3.29 (3.18-3.40)	2.83 (2.72-2.94)
Mechanical ventilation for acute infection							
No	385 210 (99.0)	367 068 (99.1)	18 142 (97.6)	4.71 (4.64-4.78)	5.19 (5.11-5.26)	1 [Reference]	1 [Reference]
Yes	3770 (1.0)	3325 (0.9)	445 (2.4)	17.36 (15.79-18.89)	18.76 (17.06-20.42)	3.66 (3.33-4.02)	1.64 (1.49-1.81)
No. of symptoms at presentation with acute infection							
0	174 818 (44.9)	167 491 (45.2)	7327 (39.4)	4.14 (4.04-4.24)	4.63 (4.53-4.74)	1 [Reference]	1 [Reference]
1-2	89 460 (23.0)	84 993 (22.9)	4467 (24.0)	5.11 (4.96-5.26)	5.59 (5.42-5.75)	1.24 (1.20-1.29)	1.10 (1.06-1.14)
3-4	60 092 (15.4)	56 849 (15.3)	3243 (17.4)	5.51 (5.32-5.70)	5.98 (5.77-6.18)	1.34 (1.28-1.39)	1.21 (1.16-1.26)
≥5	64 610 (16.6)	61 060 (16.5)	3550 (19.1)	5.46 (5.28-5.64)	5.95 (5.75-6.14)	1.33 (1.28-1.39)	1.19 (1.14-1.24)

Abbreviations: ACE, angiotensin-converting enzyme; ARB, angiotensin II receptor blocker; BMI, body mass index (calculated as weight in kilograms divided by height in meters squared); CCI, Charlson Comorbidity Index; CHF, congestive heart failure; CKD, chronic kidney disease; COPD, chronic obstructive pulmonary disease; HR, hazard ratio; *ICD-10*, *International Statistical Classification of Diseases, Tenth Revision*; PTSD, posttraumatic stress disorder.

^a^ Adjusted by Cox proportional hazards regression for age (using the categories shown), sex, race, ethnicity, urban or rural residence, CCI, and Veterans Integrated Service Network. When we evaluated the associations of any of the individual comorbidities (eg, COPD, CHF, CKD, diabetes, depression, PTSD, bipolar or schizoaffective disorder, cancer, hypertension, obesity, cerebrovascular disease, smoking, and others), we did not simultaneously adjust for the CCI because it would result in overadjustment.

^b^ Not reported for 1826 patients.

### Factors Independently Associated With U09.9 Documentation

Between October 31, 2021, and January 31, 2023, a total of 35 875 clinical encounters (in 18 587 unique patients) documented the *ICD-10* code U09.9 during a mean (SD) follow-up of 8.6 (4.7) months. Most commonly, these encounters occurred in outpatient (including telehealth) clinics, including primary care and general internal medicine (28.2%), pulmonary and respiratory therapy (11.7%), geriatrics (11.4%), telephone case management (a screening program for PCC administered via telephone in some VA facilities [11.3%]), physical therapy (6.5%), urgent care or emergency department (4.4%), occupational therapy (3.2%), mental health (2.2%), rehabilitation (2.2%), and cardiology (1.8%), with much smaller representation (<1%) in infectious diseases, neurology, and nephrology (Table 3).

**Table 3.** Distribution of Clinic Type for Encounters in 18 587 Unique Patients

Clinic type	Encounters with U09.9 documentation, No. (%) (n = 35 875)
Primary care and general internal medicine	10 131 (28.2)
Pulmonary and respiratory therapy	4193 (11.7)
Geriatrics	4089 (11.4)
Telephone case management and screening	4070 (11.3)
Physical therapy	2329 (6.5)
Urgent care or emergency department	1576 (4.4)
Occupational therapy	1165 (3.2)
Mental health	804 (2.2)
Rehabilitation	795 (2.2)
Cardiology	634 (1.8)
Infectious diseases	326 (0.9)
Neurology	116 (0.3)
Nephrology	54 (0.2)

The cumulative incidence of U09.9 documentation was 4.79% (95% CI, 4.72%-4.86%) at 6 months and 5.28% (95% CI, 5.20%-5.35%) at 12 months after infection. Factors positively associated with documentation of *ICD-10* code U09.9 (Table 1) included older age (which had a linear association with PCC risk), female sex (AHR, 1.23 [95% CI, 1.18-1.29]), Hispanic ethnicity (AHR, 1.33 [95% CI, 1.27-1.39]), high burden of comorbidities (including individual comorbidities such as COPD, diabetes, CKD, depression, and posttraumatic stress disorder), and baseline prescription of opioids and antidepressants. Indices of a more severe acute COVID-19 presentation, such as number of acute symptoms (AHR for ≥5 symptoms, 1.19 [95% CI, 1.14-1.24]), requiring hospitalization (AHR, 2.83 [95% CI, 2.72-2.94]), and requiring mechanical ventilation (AHR, 1.64 [95% CI, 1.49-1.81]) were strongly associated with higher likelihood of U09.9 documentation. Black (compared with White) race (AHR, 0.72 [95% CI, 0.69-0.75]), urban (compared with rural) residence (AHR, 0.90 [95% CI, 0.86-0.94]), primary vaccination (AHR, 0.80 [95% CI, 0.78-0.83]), and booster vaccination (AHR, 0.66 [95% CI, 0.64-0.69]) were associated with a lower likelihood of U09.9 documentation.

### Variation in U09.9 Documentation by VISN and Facility

There was great variability across VISNs in U09.9 documentation. In VISN 8 (the Sunshine Healthcare Network in Florida), there was a 12-month incidence of U09.9 documentation of 3.39% (95% CI, 3.20%-3.58%), compared with 24.90% (95% CI, 24.26%-25.53%) in VISN 17 (Heart of Texas). Compared with VISN 8, many VISNs had significantly higher likelihood of U09.9 documentation, including VISN 20 (Pacific Northwest; AHR, 2.22 [95% CI, 2.03-2.42]) and VISN 17 (AHR, 7.60 [95% CI, 7.14-8.10]). There was even greater variability by facility (medical center) ranging from less than 3% to 58.86%. (Figure). Greater U09.9 code documentation was observed in VA facilities and VISNs that had established dedicated PCC clinics and escalated even further by PCC telephone-administered screening efforts that took place at some VA facilities (captured as telephone case management visits in Table 3), for example, the 2 outlier facilities in the Figure with U09.9 documentation rates of 46.14% (San Antonio, Texas) and 58.86% (Harlingen, Texas) (both in VISN 17). Associations of race and ethnicity with U09.9 documentation are shown for the region with a dedicated screening program (VISN 17) and the rest of the VA in eTable 3 in Supplement 1.

**Figure.**
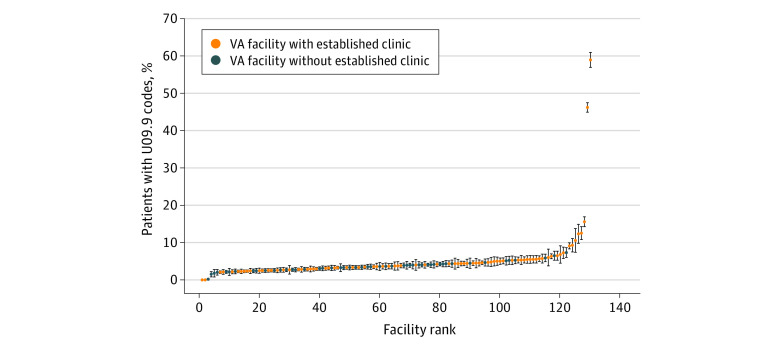
Caterpillar Plot of Documentation of Code U09.9 for Post–COVID-19 Condition (PCC) by Veterans Affairs Facility Data are expressed as proportion of patients with positive test results for SARS-CoV-2 and documented *International Statistical Classification of Diseases and Related Health Problems, Tenth Revision,* code U09.9. Veterans Affairs facilities are stratified by those in Veterans Integrated Service Networks that have established dedicated clinics for the follow-up of patients with PCC and those that do not.

### PCC Symptoms Identified by Medical Record Review in a Subset of Patients With U09.9

Among 350 randomly selected patients with the U09.9 code, 227 (64.9%) had documentation in the medical record of the presence of 1 or more new-onset symptoms present for 30 days or longer after acute COVID-19 infection. The most common symptoms were shortness of breath (130 [37.1%]), fatigue or exhaustion (78 [22.3%]), cough (63 [18.0%]), reduced cognitive function or brain fog (22 [6.3%]) and change in smell and/or taste (20 [5.7%]) (Table 4). The most common organ system involved was the respiratory system (144 [41.1%]).

**Table 4.** Symptoms Persisting for More Than 30 Days After Record of Positive Test Result^a^

Symptom type	Proportion of patients with symptom documented in their medical records, No. (%)^b^
Any	227 (64.9)
By symptom	
Dyspnea or shortness of breath	130 (37.1)
Fatigue or exhaustion	78 (22.3)
Cough	63 (18.0)
Reduced cognitive function or brain fog	22 (6.3)
Change in smell and/or taste	20 (5.7)
Abdominal pain	<5%
Headache	<5%
Fever	<5%
Chest pain	<5%
Unusual muscle pain	<5%
Diarrhea	<5%
By organ system	
Respiratory	144 (41.1)
Neurological	42 (12.0)
Musculoskeletal	<5%
Cardiovascular	38 (10.9)
Gastrointestinal tract	<5%
Dermatologic	<5%
Endocrine or metabolic	<5%
Genitourinary	<5%
Hematopoietic	<5%
Infectious diseases	<5%
Mental disorders	<5%
Sensory organs and other general symptoms	89 (25.4)

^a^ Results are from a random subsample of 350 patients with documentation of *International Statistical Classification of Diseases, Tenth Revision*, code U09.9 during the 6-month period after the positive test result for SARS-CoV-2, based on review of their medical records (including progress notes) by 2 expert clinical reviewers.

^b^ Numbers are not reported for proportions of <5%.

## Discussion

In a cohort of VA enrollees with positive test results for SARS-CoV-2 between October 1, 2021, and January 31, 2023, the cumulative incidence of code U09.9 documentation assessed across multiple health systems was 4.79% (95% CI, 4.72%-4.86%) at 6 months and 5.28% (95% CI, 5.20%-5.35%) at 12 months after infection. Older age, high comorbidity burden, female sex, and Hispanic or Latino ethnicity were independently associated with higher likelihood of U09.9 documentation, as was severity of the acute infection (manifested by symptoms, hospitalization, or ventilation). Black (compared with White) race and urban (compared with rural) residence were associated with a lower likelihood, as were primary and booster vaccination (compared with no vaccination). Very marked differences by VISN and facility in U09.9 code documentation reflected local practices of screening and care for PCC. The most common symptoms recorded in the medical record in patients with documented U09.9 code were shortness of breath, fatigue, cough, reduced cognitive function and change in smell and/or taste.

In an analysis published earlier in the pandemic (July 2022), Ioannou et al^22^ reported a higher rate of PCC documented among veterans (13.5%) compared with 5.28% in the current analysis. Differences between these studies likely contribute to the dissimilarities seen. First, in the earlier analysis, 88% of participants had no SARS-CoV-2 vaccine doses recorded compared with only 26.8% of the current cohort. Second, the earlier analysis used a list of several diagnosis codes to identify PCC (U07.1, Z86.16, U09.9, and J12.82). This approach was used because although *ICD-10* code U09.9 is specific for PCC, it was not introduced until October 1, 2021. Third, evolving SARS-CoV-2 variants may act differently to contribute to the development of PCC due to differences in infectivity, immune system evasion, or other factors^23^; we were unable to examine this possibility because our study period encompassed almost exclusively the Omicron-predominant era.

A population-based study from the 2 largest regions of Sweden (n = 4.1 million inhabitants, where code U09.9 was implemented in October 2020, 1 year earlier than in the US)^24^ reported that among 506 107 registered cases of COVID-19, only 10 196 (2.0%) had documentation of U09.9 as of February 15, 2022. This lower proportion compared with what we report herein might be related to differences in study design and study population, as Swedish participants were younger and had a lower burden of comorbidities. The Swedish study reported higher rates of U09.9 documentation in hospitalized vs nonhospitalized patients, women vs men, patients aged 55 to 64 years vs younger or older age groups, those infected during Alpha vs Delta variant predominance, and those with prior respiratory disease, cardiovascular disease, or diabetes, which are broadly similar to risk factors noted in our study. The National Institutes of Health (NIH) National COVID Cohort Collaborative (N3C) study reported on 33 782 patients from 34 different health care systems with 1 or more U09.9 codes documented from October 1, 2021, to May 26, 2022.^25^ However, the N3C study did not include a comparison population of infected patients without U09.9 documentation. Hence, they did not report factors directly associated with U09.9 documentation.

We found that primary and booster vaccination against SARS-CoV-2 infection was associated with a lower likelihood of U09.9 documentation. To our knowledge, no randomized clinical trials have assessed the effect of vaccination to prevent or treat PCC. The findings of our study are generally consistent with those of several previous observational studies on the topic^26,27^ and an observational study emulating a target trial of PCC vaccination.^28^ Given the accumulated observational evidence and the low risk of vaccination, clinicians should recommend primary and booster vaccination to patients interested in reducing their risk of PCC.

Our systematic medical record review of 350 individuals with U09.9 documentation revealed that 64.9% had new-onset symptoms thought to be related to COVID-19 that persisted for 30 days or more, but the remaining 35.1% did not. Common reasons for this were that symptoms documented in the first 30 days did not persist beyond 30 days or that patients were assigned the U09.9 code for exacerbations of preexisting conditions rather than new-onset symptoms. The most common symptoms documented in the medical record of VA patients with the U09.9 code was shortness of breath, followed by fatigue, cough, reduced cognitive function, and change in smell and/or taste. This pattern was in broad agreement with the symptoms listed in the World Health Organization definition of post–COVID-19 condition, which particularly highlights “fatigue, shortness of breath and cognitive dysfunction”^29^ and also with a scoping review of the literature on PCC.^30^ However, a recent analysis^31^ based on the NIH’s RECOVER (Researching COVID to Enhance Recovery) cohort developed a definition and scoring system for postacute sequelae of SARS-CoV-2 infection (PASC) based on symptoms more commonly present in infected vs uninfected participants 6 months after infection, including the following symptoms ordered by decreasing frequency: postexertional malaise, fatigue, reduced cognitive function, dizziness, gastrointestinal tract symptoms, palpitations, sexual symptoms, loss or change in smell or taste, thirst, cough, chest pain, and abnormal movements. The main difference is the predominance of respiratory symptoms in our study, which feature less prominently in the NIH-RECOVER PASC model.

We observed substantial differences in rates of U09.9 documentation by region and facility. Although our study was not designed specifically to investigate such differences, we can speculate about factors underlying this pattern. First, the presence of a PCC clinic is likely expected to increase U09.9 documentation rates because these specialized clinicians have greater familiarity with the use of the code. Consistent with this, facilities within VISNs that have dedicated PCC clinics tended to have higher rates of U09.9 code documentation than facilities in VISNs without PCC clinics (Figure). Second, we observed 2 facilities with comparatively high U09.9 use, both in VISN 17. This was directly related to the establishment of telephone-based screening of all persons with positive test results for SARS-CoV-2 and symptoms that might be related to PCC at these 2 sites. Additional factors may have also contributed to the regional differences in U09.9 coding rates such as regional awareness, education, cultural and political influences, and regional variation in the risk factors for PCC. In the future, accurate and consistent documentation of U09.9 is needed to maximize its utility in tracking patients for clinical care and research.

### Strengths and Limitations

This analysis has several strengths, most notably the use of a large, well-characterized, national analytic cohort, with representation of diverse races and ethnicities, electronic records harmonized across the VA EHR, VA Community Care program, and CMS-Medicare for U09.9 ascertainment, and structured medical record extraction for PCC symptoms. There are also some limitations. Most importantly, the U09.9 code is a proxy for the presence of PCC and codes may be used inconsistently especially in the early adoption period or may be underused in populations with limited access to care. Next, the analysis included a relatively small proportion of women (12.7% of the cohort); however, given the large sample size, almost 50 000 women were included in the analysis. Finally, findings may not be broadly generalizable outside the VA health care population, which is on average older and has lower socioeconomic status than the general US population.^32^

## Conclusions

In this large national cohort study that included US Veterans with positive test results for SARS-CoV-2 between October 1, 2021, and January 31, 2023, the cumulative incidence of U09.9 documentation was 5.28% at 12 months. Older age, female sex, Hispanic or Latino ethnicity, hospitalization, receipt of mechanical ventilation, and a higher number of symptoms at presentation were associated with a higher likelihood of U09.9 documentation, while vaccination was associated with a lower likelihood. Nearly two-thirds of individuals with U09.9 documentation had symptoms for 30 days or longer, most commonly respiratory symptoms. Marked differences were seen across geographic regions and facilities. Future studies should examine the long-term trajectory of individuals with U09.9 documentation.

## Notes

Supplement 1. **eTable 1.** Yes/No Flags for Common COVID-19 Symptoms in the 30 Days Prior to the Index Date Extracted From the VHA EHR **eTable 2.** Selected Baseline Characteristics of the SARS-CoV-2–Positive Patients in the Primary Study Cohort and in the Medical Record Review Cohort **eTable 3.** Associations of Race and Ethnicity With Risk of U09.9 Documentation, Stratified by Region (VISN 17 vs not VISN 17)

Supplement 2.
Data Sharing Statement
