# Supplementary material for: Rates of ICD-10 Code U09.9 Documentation and Clinical Characteristics of VA Patients With Post–COVID-19 Condition
Source: JAMA Netw Open. 2023 Dec 8;6(12):e2346783. doi: 10.1001/jamanetworkopen.2023.46783 (PMC10709773; doi:10.1001/jamanetworkopen.2023.46783)
Supplement: Supplement 1. — eTable 1. Yes/No Flags for Common COVID-19 Symptoms in the 30 Days Prior to the Index Date Extracted From the VHA EHR eTable 2. Selected Baseline Characteristics of the SARS-CoV-2–Positive Patients in the Primary Study Cohort and in the Medical Record Review Cohort eTable 3. Associations of Race and Ethnicity With Risk of U09.9 Documentation, Stratified by Region (VISN 17 vs not VISN 17) [file jamanetwopen-e2346783-s001.pdf]

Supplemental Online Content

Wander PL, Baraff A, Fox A, et al. Rates of *ICD-10* code U09.9 documentation and clinical characteristics of VA patients with post–COVID-19 condition. *JAMA Netw Open*. 2023;6(12):e2346783. doi:10.1001/jamanetworkopen.2023.46783

eTable 1. Yes/No Flags for Common COVID-19 Symptoms in the 30 Days Prior to the Index Date Extracted From the VHA EHR
eTable 2. Selected Baseline Characteristics of the SARS-CoV-2–Positive Patients in the Primary Study Cohort and in the Medical Record Review Cohort
eTable 3. Associations of Race and Ethnicity With Risk of U09.9 Documentation, Stratified by Region (VISN 17 vs not VISN 17)

This supplemental material has been provided by the authors to give readers additional information about their work.

© 2023 Wander PL et al. *JAMA Network Open.*

**eTable 1.** Yes/No Flags for Common COVID-19 Symptoms in the 30 Days Prior to the Index Date Extracted From the VHA EHR
Abdominal pain
Chills
Cold symptoms
Cough
Diarrhea
Dyspnea
Fatigue
Fever
Headache
Loss of smell
Loss of taste
Myalgias
Nausea
Rhinorrhea
Sore throat

© 2023 Wander PL et al. *JAMA Network Open.*

eTable 2. Selected Baseline Characteristics of the SARS-CoV-2–Positive Patients in the Primary Study Cohort and in the Medical Record Review Cohort

Demographics	All SARS-CoV-2–positive patients in the primary study cohort	SARS-CoV-2–positive* patients with a U09.9 ICD code sampled for the medical record review
Total	18587	350
Age at incident COVID Diagnosis Mean (SD)	61.4	63.4 (14.06)
**Sex**		
Male	16178 (87.0%)	310 (88.83%)
Female	2409 (13.0%)	39 (11.17%)
**Race**		
African-American or Black	2755 (21.0%)	66 (18.9%)
American Indian or Alaska Native	191 (1.0%)	3 (0.9%)
Asian	193 (1.0%)	3 (0.9%)
Native Hawaiian or Pacific Islander	198 (1.1%)	6 (1.7%)
White	13541 (72.9%)	247 (70.8%)

© 2023 Wander PL et al. *JAMA Network Open.*

eTable 3. Associations of Race and Ethnicity With Risk of U09.9 Documentation, Stratified by Region (VISN 17 vs not VISN 17)

Race	In VISN 17 Hazard Ratio (95% CI) Crude	In VISN 17 Hazard Ratio (95% CI) Adjusted*	Not in VISN 17 Hazard Ratio (95% CI) Crude	Not in VISN 17 Hazard Ratio (95% CI) Adjusted*	p from interaction model
African American or Black	0.51 (0.47, 0.56)	0.61 (0.56, 0.67)	0.70 (0.67, 0.74)	0.77 (0.74, 0.81)	<0.0001
American Indian or Alaska Native	1.11 (0.86, 1.43)	1.01 (0.77, 1.34)	1.12 (0.94, 1.32)	1.12 (0.95, 1.33)	0.524
Asian	0.65 (0.48, 0.88)	0.90 (0.66, 1.24)	0.71 (0.61, 0.83)	0.93 (0.79, 1.09)	0.882
Native Hawaiian or Pacific Islander	0.71 (0.55, 0.91)	0.72 (0.55, 0.95)	0.96 (0.81, 1.13)	1.08 (0.91, 1.28)	0.013
White	1.00	1.00	1.00	1.00	
Declined or Missing	0.93 (0.86, 1.01)	0.96 (0.88, 1.05)	0.91 (0.85, 0.96)	1.01 (0.94, 1.08)	0.368
**Ethnicity**					
Not Hispanic or Latino	1.00	1.00	1.00	1.00	
Hispanic or Latino	1.79 (1.69, 1.90)	1.90 (1.78, 2.02)	0.80 (0.75, 0.86)	0.89 (0.83, 0.96)	<0.0001
Declined or Missing	1.06 (0.95, 1.18)	1.10 (0.98, 1.24)	0.85 (0.79, 0.92)	0.91 (0.84, 0.98)	0.007

© 2023 Wander PL et al. *JAMA Network Open.*
