# Supplementary material for: Rates of ICD-10 Code U09.9 Documentation and Clinical Characteristics of VA Patients With Post–COVID-19 Condition
Source: JAMA Netw Open. 2023 Dec 8;6(12):e2346783. doi: 10.1001/jamanetworkopen.2023.46783 (PMC10709773; doi:10.1001/jamanetworkopen.2023.46783)
Supplement: Supplement 2. — Data Sharing Statement [file jamanetwopen-e2346783-s002.pdf]

Data Sharing Statement

Wander. Rates of ICD-10 Code U09.9 Documentation and Clinical Characteristics of VA Patients With Post–COVID-19 Condition. *JAMA Netw Open*. Published December 08, 2023. doi:10.1001/jamanetworkopen.2023.46783

Data
Data available: No

Additional Information
Explanation for why data not available: Data cannot be shared outside VA.
